# Low-frequency oscillations in the brain show differential regional associations with severity of cerebral small vessel disease: a systematic review

**DOI:** 10.3389/fnins.2023.1254209

**Published:** 2023-08-31

**Authors:** James Thomas, Peter Jezzard, Alastair J. S. Webb

**Affiliations:** ^1^Nuffield Department of Clinical Neurosciences, Wolfson Centre for Prevention of Stroke and Dementia, John Radcliffe Hospital, University of Oxford, Oxford, United Kingdom; ^2^FMRIB Division, Nuffield Department of Clinical Neurosciences, Wellcome Centre for Integrative Neuroimaging, John Radcliffe Hospital, University of Oxford, Oxford, United Kingdom

**Keywords:** low-frequency oscillations, cerebral small vessel disease, neuroimaging markers, endothelial dysfunction, fMRI

## Abstract

**Background:**

Cerebral small vessel disease (cSVD) is associated with endothelial dysfunction but the pathophysiology is poorly understood. Low-frequency oscillations (LFOs) in the BOLD signal partly reflect cerebrovascular function and have the potential to identify endothelial dysfunction in cSVD. A systematic review was performed to assess the reported relationships between imaging markers of cSVD and LFOs.

**Methods:**

Medline and EMBASE were searched for original studies reporting an association between LFOs and STRIVE-defined imaging markers of cSVD, including: white matter hyperintensities (WMH), enlarged perivascular spaces, lacunes, CADASIL, and cerebral microbleeds, from inception to September 1, 2022. Variations in LFOs were extracted, where available, on a global, tissue-specific, or regional level, in addition to participant demographics, data acquisition, methods of analysis, and study quality. Where a formal meta-analysis was not possible, differences in the number of studies reporting LFO magnitude by presence or severity of cSVD were determined by sign test.

**Results:**

15 studies were included from 841 titles. Studies varied in quality, acquisition parameters, and in method of analysis. Amplitude of low-frequency fluctuation (ALFF) in resting state fMRI was most commonly assessed (12 studies). Across 15 studies with differing markers of cSVD (9 with WMH; 1 with cerebral microbleeds; 1 with lacunar infarcts; 1 with CADASIL; 3 with multiple markers), LFOs in patients with cSVD were decreased in the posterior cortex (22 of 32 occurrences across all studies, *p* = 0.05), increased in the deep grey nuclei (7 of 7 occurrences across all studies, *p* = 0.016), and potentially increased in the temporal lobes (9 of 11 occurrences across all studies, *p* = 0.065).

**Conclusion:**

Despite limited consensus on the optimal acquisition and analysis methods, there was reasonably consistent regional variation in LFO magnitude by severity of cSVD markers, supporting its potential as a novel index of endothelial dysfunction. We propose a consistent approach to measuring LFOs to characterise targetable mechanisms underlying cSVD.

## Introduction

Chronic ischaemic injury to the small vessels in the brain (cerebral small vessel disease, cSVD), is associated with up to 30% of strokes and 40% of dementia (Inzitari et al., 2009; Shi and Wardlaw, 2016), but has no specific treatment. It is defined on MRI of the brain by white matter hyperintensities (WMHs), enlarged perivascular spaces, cerebral microbleeds, and lacunar infarcts, which are agreed to be imaging manifestations of a common small vessel vasculopathy (Wardlaw et al., 2013b). cSVD is particularly associated with long-term hypertension from mid-life (Wartolowska and Webb, 2021), as well as diabetes, smoking, and genetic factors. Furthermore, severity of cSVD is associated with markers of cerebrovascular endothelial dysfunction, including blood–brain barrier breakdown and impaired cerebrovascular reactivity to either neuronal activity (Dumas et al., 2012; Banerjee et al., 2017) or inhaled carbon dioxide (Zupan et al., 2015). However, there is no currently available index of cerebrovascular endothelial dysfunction applicable in large epidemiological studies or routine clinical practice.

Low-frequency oscillations (LFOs) are rhythmic oscillations in imaging markers of cerebral blood flow or volume, usually defined in a frequency range from 0.008–0.15 Hz. The most commonly measured index of LFOs is the amplitude of low-frequency fluctuation (ALFF), defined as the average square root of the voxel-wise power spectrum of BOLD-MRI within a given frequency range (Zang et al., 2007). The BOLD signal is a measurement of haemodynamic properties of blood flow, volume, and oxygenation changes (Buxton et al., 1998). ALFF can be divided by the global power within the entire detectable frequency to give fractional ALFF (fALFF), which can minimise the impact of global changes in variability in blood flow in the low-frequency band. Though fALFF mitigates the influence of non-neuronal physiological “noise” and enables better differentiation of signals generated from grey matter versus white matter, it can be more sensitive to fMRI preprocessing steps and normalisation could potentially result in diminished signal-to-noise ratio (Zou et al., 2008). ALFF has been principally investigated as a marker of neuronal activity in conditions including Alzheimer’s disease (Yang et al., 2018), Parkinson’s disease (Yue et al., 2020), and mild cognitive impairment (Xi et al., 2012; Pan et al., 2017). However, similar rhythmic oscillations in blood flow are seen in multiple organs, reflecting autonomic control of blood flow. These are seen in blood pressure, baroreceptor function, heart rate variability, and spontaneous cerebral autoregulation (deBoer et al., 1987; Cooley et al., 1998; Julien, 2006) and likely reflect synchronised oscillations in endothelial tone under the influence of the sympathetic nervous system (Bernardi et al., 1997), often referred to as vasomotion. Brain imaging studies usually use 0.1 Hz as an upper limit for measuring LFOs, but these systemic oscillations are more commonly divided into very-low-frequency (0.01–0.04 Hz), low-frequency (0.04–0.15 Hz), and high-frequency (0.15–0.45 Hz; Francis et al., 2000), due to endogenously determined peaks in rhythmic oscillations at approximately 0.1 Hz and 0.25 Hz.

We therefore hypothesised that altered amplitude of LFOs in BOLD signal may reflect underlying endothelial dysfunction as a potential marker of cSVD, applicable in large epidemiological cohorts and clinical practise. We performed a systematic review of previous imaging studies to assess the reported relationship between classical markers of cSVD and altered amplitude of LFOs.

## Methods

### Search strategy

We searched EMBASE and Medline for studies published in English from inception to September 1, 2022 to identify all original studies reporting an association between cerebral SVD and LFOs. The search was conducted as follows: [“(s)LFO(s)” OR “(spontaneous) low-frequency oscillation(s)” OR “(fractional) amplitude of low frequency fluctuation(s)” OR “(f)ALFF” OR “mayer(s)” OR “low-frequency fluctuation(s)” OR “low-frequency power”] AND [“small vessel disease” OR “SVD” OR “CSVD” OR “leukoaraiosis” OR “leucoaraiosis” OR “white matter hyperintensity (ies)” OR “WMH(s)” OR “white matter lesion(s)” OR “WML(s)” OR “perivascular space(s)” OR “microbleed(s)” OR “subcortical infarct(s)” OR “subcortical infarction(s)” OR “lacune(s)” OR “lacunar infarct(s)” OR “lacunar infarction(s)” OR “CADASIL”] (Supplementary Table S1).

All study titles and abstracts were reviewed and those that potentially met the eligibility criteria were screened in full. The reference lists of all identified articles were screened to identify any potentially missed studies. The included studies were quality assessed using the NIH Quality Assessment Tool for Observational Cohort and Cross-Sectional Studies. All included papers are in published journals.

### Study selection

Studies had to include a measurement of absolute low-frequency power in the 0.008–0.15 Hz frequency band on magnetic resonance imaging (MRI) or near-infrared spectroscopy (NIRS) with a monogenic SVD (e.g., CADASIL) or sporadic small vessel disease defined by the cardinal MRI-based imaging features-WMHs (also known as leukoaraiosis), enlarged perivascular spaces, lacunes, and cerebral microbleeds-as per the consensus STandards for ReportIng Vascular changes on nEuroimaging (STRIVE) criteria for definition of cSVD (Wardlaw et al., 2013b). Only original peer-reviewed studies were included. Studies in animals, children, conference abstracts, and those not available in English were excluded (Supplementary Table S2).

### Data extraction

From each study, we extracted data on: (1) the population (number of participants, inclusion of controls, mean age of all participants, and criteria for diagnosing cSVD); (2) the features of data acquisition (imaging modality, scanner field strength, repetition time, echo time, scan duration), and (3) the methods of analysis (the analytical method of derivation of LFOs, frequency-band studied, normalisation of values, and use of spatial smoothing). *A priori*, we extracted data reporting an index of LFOs for the whole brain and by tissue type as a likely global index of systemic LFOs in blood flow into the brain and physiological determinants of endothelial function, independent of neuronal activity. Secondly, we extracted data on regional variation in LFOs.

### Statistical analysis

Where more than 5 papers reported a consistent quantitative result of LFOs in a comparable population, a meta analysis was performed weighted by inverse variance. Where this was not possible, the frequency of direction of effect was assessed by the sign test. This was performed for all occurrences when a study noted multiple changes in LFOs within the same cortical region.

## Results

**Figure 1:**
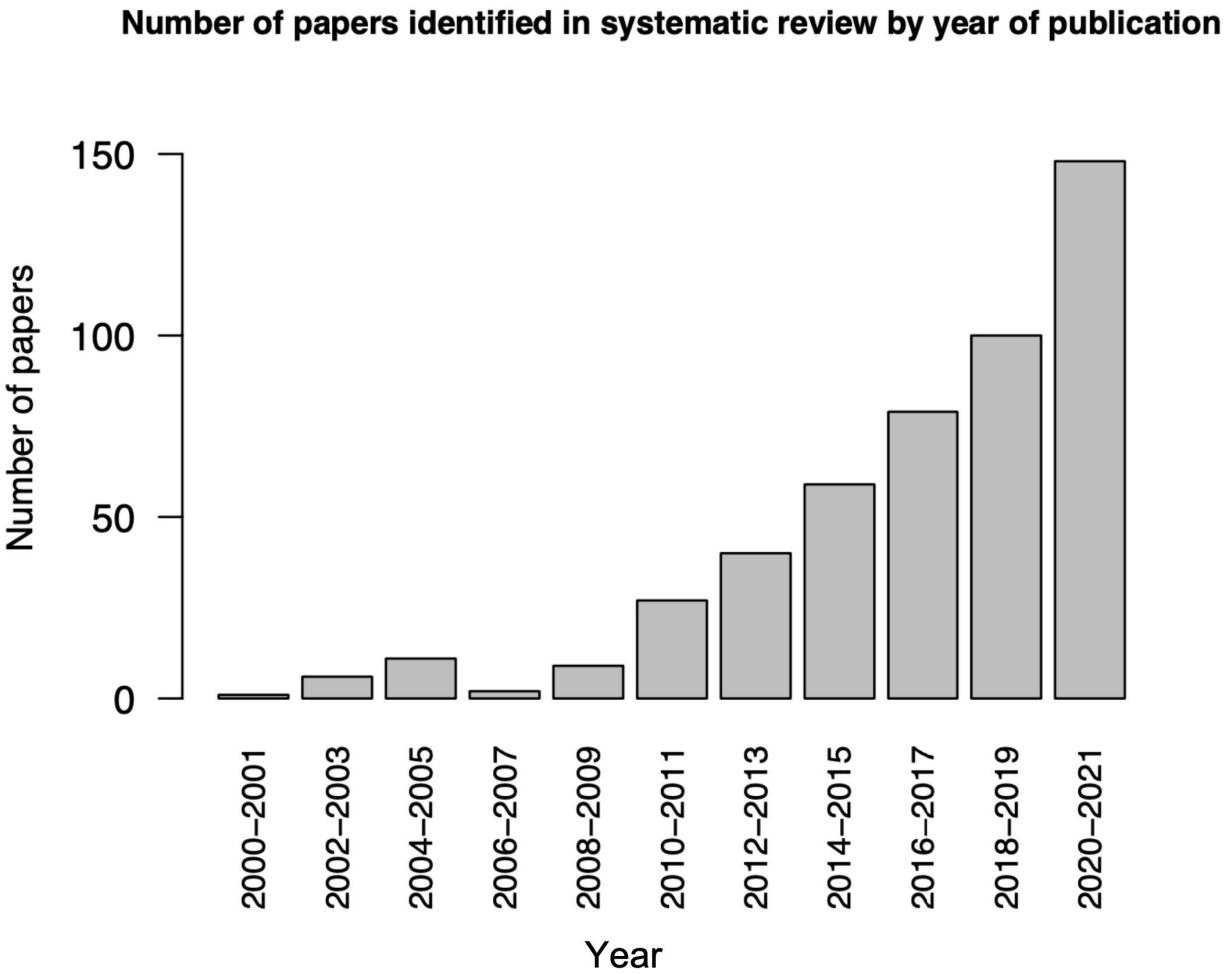
The number of papers identified in this systematic review by year of publication.

**Figure 2:**
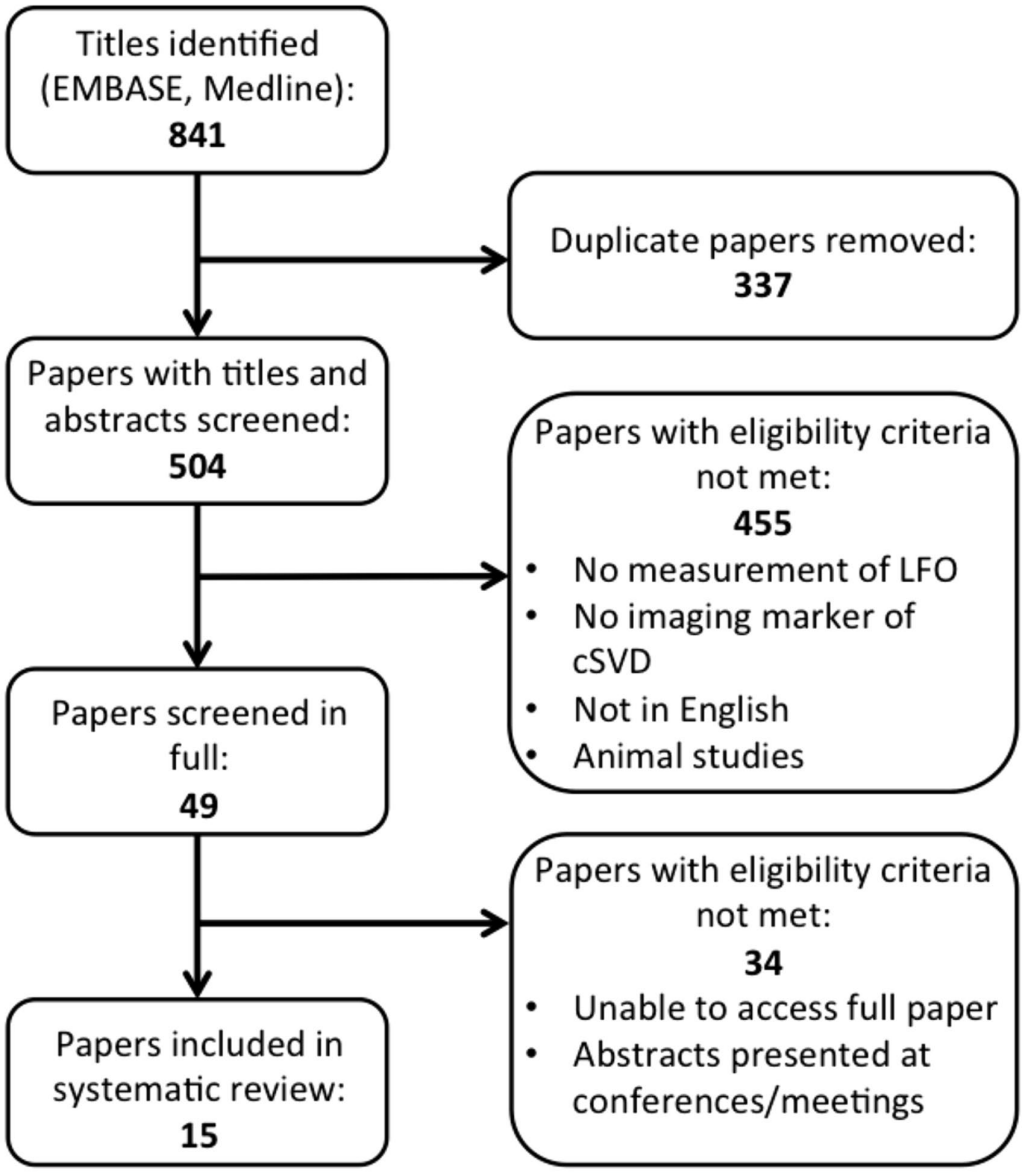
PRISMA diagram for search.

Of 841 titles identified, 504 studies remained after removal of duplications, with a significant increase in the number of publications on LFOs published per year in the last decade (Figure 1). After review of abstracts and full text, 15 studies were included (Figure 2). The main reasons for exclusion included: no measurement of cSVD; no index of LFOs; animal studies; papers not published in English; and abstracts presented at conferences.

### Quality of the studies

Two of the studies were graded as “good quality,” seven were graded as “fair quality,” and six were graded as “poor quality” on the NIH Quality Assessment Tool for Observational Cohort and Cross-Sectional Studies (Supplementary Table S3). The main reasons studies were graded as poor quality were: the use of multiple comparisons with no statistical correction; no sample size justification; and minimal or no adjustment for potentially confounding variables.

### Acquisition and analysis methods of the studies

There was reasonable consistency between studies in the method of data acquisition, with all studies deriving LFOs from resting state fMRI on 3 Tesla scanners, except for one that used NIRS. Ten studies used a Repetition Time (TR) of 2000 ms, one 2,400 ms, one 1,400 ms, one 1,500 ms, and one 3,000 ms. All but one study used an Echo Time (TE) of 30 ms. The frequency band analysed was relatively consistent, with nine of the MRI studies using the frequency band 0.01–0.08 Hz (Table 1). Few studies reported differences in LFOs by severity of cSVD in the higher frequency bands (>0.12 Hz).

**Table 1:** Summary of extracted data for all eligible studies.

Imaging marker of cSVD	Author (year)	Patients	Controls	Measure of LFOs	Frequency range (Hz)	ROIs with increased LFOs in patients compared to controls	ROIs with decreased LFOs in patients compared to controls
WMHs	Cao et al. (2021)	74	35	ALFF	0.01–0.1	Bilateral thalamus	No associations reported
WMHs	Cheng et al. (2017)	28	30	ALFF	0.01–0.08	Left inferior semi-lunar lobule; right superior orbital frontal gyrus	Left parahippocampal gyrus
WMHs	Ding et al. (2015)	22	12	fALFF	0.01–0.08	No associations reported	No associations reported
WMHs	Kim et al. (2020)	17	21	ALFF	0.009–0.08	No associations reported	No associations reported
WMHs	Li et al. (2015)	56	28	ALFF	0.01–0.08	Right inferior occipital gyrus; left precuneus; right superior frontal gyrus; right superior occipital gyrus	Left middle temporal gyrus
WMHs	Ni et al. (2022)	111	72	ALFF	0.01–0.08	Temporal areas; frontal areas; parietal areas	Frontal areas; precuneus/posterior cingulate cortex; parietal areas
WMHs	Porcu et al. (2021)	75	0	fALFF	0.008–0.09	No associations reported	Prefrontal cortex; precuneus; cerebellar crus I/II
WMHs	Wang et al. (2019)	46	28	ALFF	0.01–0.08	Temporal regions; right inferior temporal gyrus	Posterior cingulate cortex/precuneus
WMHs	Xing et al. (2022)	69	0	ALFF	0.01–0.08	Right middle frontal gyrus	Left middle occipital gyrus
CMBs	Feng et al. (2021)	66	36	fALFF, ALFF	0.01–0.08	Right insula; putamen; left precuneus	Right precentral gyrus; postcentral gyrus
CADASIL	Su et al. (2019)	22	44	ALFF	0.01–0.08	Bilateral superior frontal gyrus; left cerebellar anterior and posterior lobes	Right precuneus and cuneus
LIs	Ni et al. (2016)	48	28	ALFF	0.01–0.08	Temporal lobe	Precuneus/cuneus; frontal lobe
LIs and/or WMHs	Yi et al. (2012)	26	28	ALFF	0.01–0.1	Posterior part of the DMN (i.e., the posterior cingulate/precuneus); right hippocampus; right thalamus	Anterior part of the DMN (i.e., the medial prefrontal cortex)
SIVD	Li et al. (2014)	30	35	ALFF	0.01–0.073	Bilateral anterior cingulate cortex; right putamen; right supplementary motor area	Right precuneus; right angular gyrus
LIs and/or PWMLs	Schroeter et al. (2005)	13	14	LFOs on NIRS	0.018–3	No associations reported	Visual cortex

cSVD, cerebral small vessel disease; LFO, lof-frequency oscillations; ROIs, regions of interest; WMHs, white matter hyperintensities; ALFF, amplitude of low-frequency fluctuations; fALFF, fractional amplitude of low-frequency fluctuations; NIRS, near-infrared spectroscopy; CMBs, cerebral microbleeds; LIs, lacunar infarcts; SIVD, subcortical ischemic vascular disease; PWMLs, periventricular white matter lesions; CADASIL, Cerebral Autosomal Dominant Arteriopathy with Sub-cortical Infarcts and Leukoencephalopathy; DMN, default mode network.

For analysis, 12 studies used ALFF and two used fALFF as a measure of LFOs (Table 2). Nine studies used 6 mm of spatial smoothing, three used 8 mm, one used 4 mm, and one used 20 mm. Eight of the studies normalised the individual voxel’s ALFF by dividing it by the participant’s average global ALFF. A variety of pre-processing methods were used including: principal component analysis (PCA); removal of average signals from white matter and cerebrospinal fluid (CSF) in three studies; and motion correction in 13 studies.

**Table 2:** Technical aspects of studies.

Imaging marker of cSVD	Author (year)	Measure of LFOs	Frequency range (Hz)	Spatial smoothing (mm)	Normalisation	TR (ms)	TE (ms)	Scan duration (s)
WMHs	Cao et al. (2021)	ALFF	0.01–0.1	8	Divide voxel’s ALFF by global mean ALFF	2,400	30	Not stated
WMHs	Cheng et al. (2017)	ALFF	0.01–0.08	6	Divide voxel’s ALFF by global mean ALFF	2000	30	470
WMHs	Ding et al. (2015)	fALFF	0.01–0.08	6	(fALFF-global mean fALFF)/sd of global mean power spectrum density	2000	30	460
WMHs	Kim et al. (2020)	ALFF	0.009–0.08	20	None reported	3,000	30	Not stated
WMHs	Li et al. (2015)	ALFF	0.01–0.08	6	Divide voxel’s ALFF by global mean ALFF	2000	30	480
WMHs	Ni et al. (2022)	ALFF	0.01–0.08	6	Divide voxel’s ALFF by global mean ALFF	2000	30	480
WMHs	Porcu et al. (2021)	fALFF	0.008–0.09	8	None noted	1,400	30	920
WMHs	Wang et al. (2019)	ALFF	0.01–0.08	8	Divide voxel’s ALFF by global mean ALFF	2000	30	480
WMHs	Xing et al. (2022)	ALFF	0.01–0.08	6	Divide voxel’s ALFF by global mean ALFF	2000	30	360
CMBs	Feng et al. (2021)	fALFF, ALFF	0.01–0.08	4	Normalised by DARTEL tool	1,500	30	Not stated
CADASIL	Su et al. (2019)	ALFF	0.01–0.08	6	Average ALFF subtracted from each voxel’s ALFF and divided by standard deviation of whole-brain ALFF	2000	30	420
LIs	Ni et al. (2016)	ALFF	0.01–0.08	6	ALFF of each voxel divided by global mean ALFF value	2000	30	487
Lis and/or WMHs	Yi et al. (2012)	ALFF	0.01–0.1	6	ALFF of each voxel divided by global mean ALFF value in grey matter mask	2000	40	478
SIVD	Li et al. (2014)	ALFF	0.01–0.073	6	ALFF of each voxel divided by global mean value	2000	30	Not stated
LIs and/or PWMLs	Schroeter et al. (2005)	LFOs on NIRS	0.08–3	n/a	Power spectral density normalised for every participant to 1 (integral normalisation)	n/a	n/a	n/a

cSVD, cerebral small vessel disease; LFO, lof-frequency oscillations; ROIs, regions of interest; WMHs, white matter hyperintensities; ALFF, amplitude of low-frequency fluctuations; fALFF, fractional amplitude of low-frequency fluctuations; NIRS, near-infrared spectroscopy; CMBs, cerebral microbleeds; LIs, lacunar infarcts; SIVD, subcortical ischemic vascular disease; PWMLs, periventricular white matter lesions; CADASIL, Cerebral Autosomal Dominant Arteriopathy with Sub-cortical Infarcts and Leukoencephalopathy; DMN, default mode network.

### Differences in LFOs by cSVD

**Figure 3:**
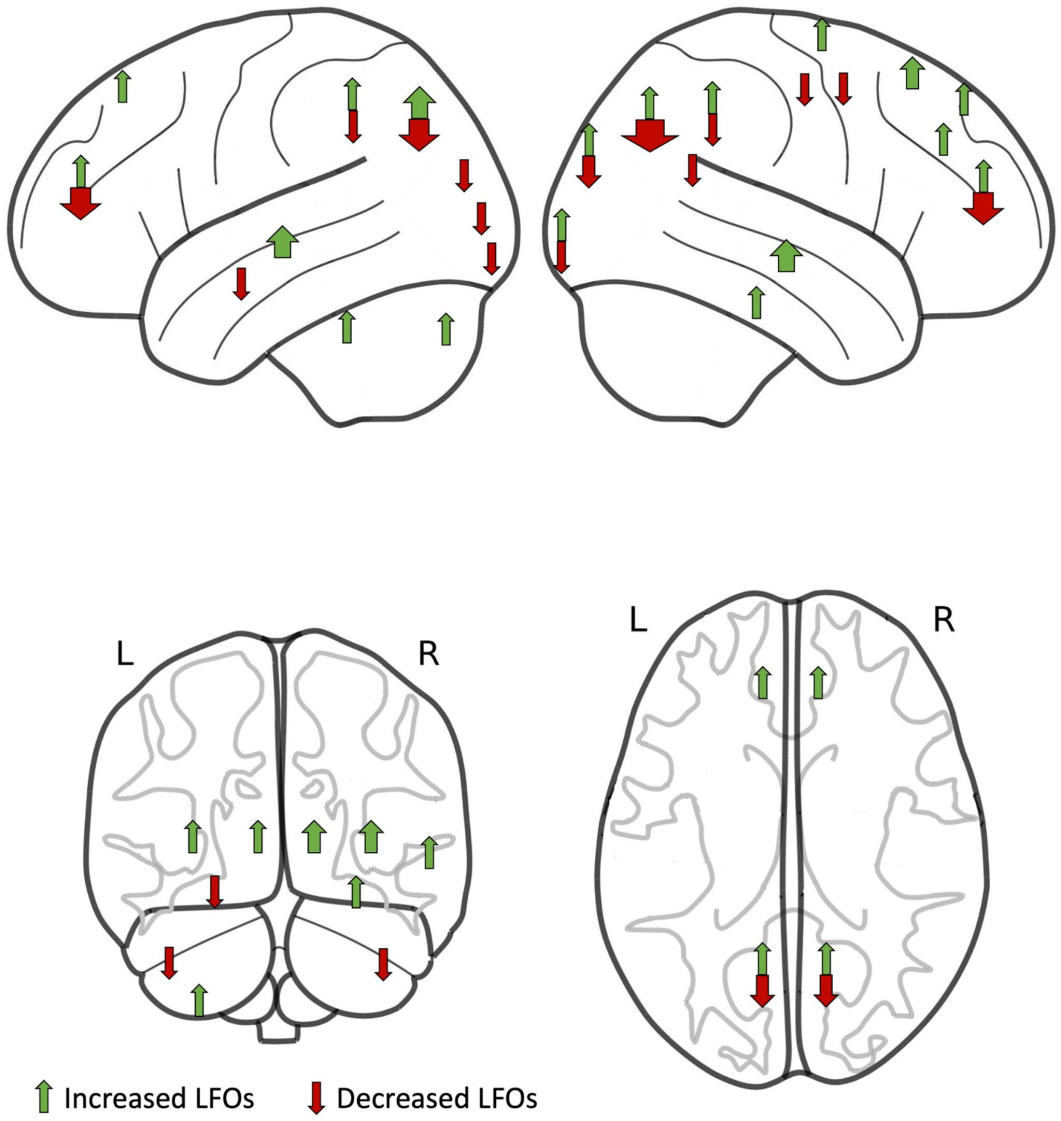
Regions of interest with increased low-frequency oscillations (LFOs) in patients compared to controls (green arrows) and with decreased LFOs in patients compared to controls (red arrows) identified by studies in this systematic review. The width of each arrow is proportional to the number of studies.

Although there were no consistent quantitative measures of LFOs reported in consistent brain regions and patient groups among studies, preventing a formal meta-analysis, there were significant and consistent differences in the direction of association between LFOs magnitude and severity of cSVD in specific brain regions (Figure 3). LFOs were consistently increased in the temporal lobe (9 of 11 occurrences across all studies, *p* = 0.065) and deep grey nuclei (7 of 7 occurrences across all studies, *p* = 0.016), and decreased in the posterior cortex (22 of 32 occurrences across all studies, *p* = 0.05) (Table 3), but without a consistent pattern in other cortical regions.

**Table 3:** Number of times differences in low-frequency oscillations are recorded by region of the brain with value of *p*s calculated from the sign test.

Region of the brain	Number of times LFOs are identified as increased in patients compared to controls	Number of times LFOs are identified as decreased in patients compared to controls	Value of *p*
Anterior cortex	10	9	1.0
Posterior cortex	10	22	0.05
Temporal cortex	9	2	0.065
Deep grey structures	7	0	0.016

LFOs, low-frequency oscillations.

### White matter hyperintensities

Nine studies reported LFOs in participants with WMHs. Five of these studies were published within the past 2 years and all were cross-sectional in design. The average number of patients and controls was 52 (range 17–111) and 29 (range 0–72), respectively. All patients and controls had a mean age of 60–70 years old. Overall, seven studies found regions of interest (ROIs) in the grey matter that had either an increase or decrease in LFOs when comparing participants with WMHs to controls (Table 1). None of the studies reported global or tissue-specific LFOs.

LFOs were elevated in deep grey structures in patients with WMHs compared to controls (Cao et al., 2021). Cao et al. reported the ALFF in 74 patients with WMHs (33 categorised as mild WMHs, with a Fazekas score 1–2, and 41 categorised as moderate–severe, with a Fazekas score of 3–6). They found increased ALFF in the bilateral thalamus of patients with mild and moderate–severe WMHs when compared with healthy controls.

LFOs were reported to be both increased or decreased in temporal brain regions in patients with WMHs compared to controls (Li et al., 2015; Cheng et al., 2017; Wang et al., 2019; Ni et al., 2022). These included significantly decreased ALFF in the left parahippocampal gyrus in 28 patients with subcortical WMHs compared to 30 healthy controls (Cheng et al., 2017) and decreased ALFF in the left middle temporal gyrus in 56 patients with mild–severe leukoaraiosis compared to 28 healthy controls (Li et al., 2015). ALFF was significantly increased in temporal regions in 111 participants with WMHs compared to 72 controls matched for age, sex, and vascular risk factors (Ni et al., 2022) and in 46 patients with leukoaraiosis when compared to 28 healthy controls (Wang et al., 2019).

The amplitude of LFOs was altered in posterior brain regions in participants with WMHs in five studies (Li et al., 2015; Wang et al., 2019; Porcu et al., 2021; Ni et al., 2022; Xing et al., 2022). LFOs were increased in participants with WMHs compared to controls in the parietal lobes (Ni et al., 2022) and in the right inferior occipital gyrus, left precuneus, and right superior occipital gyrus (Li et al., 2015). LFOs were decreased in posterior brain regions in participants with WMHs in the precuneus (Wang et al., 2019; Porcu et al., 2021; Ni et al., 2022), parietal lobes (Ni et al., 2022), and left middle occipital gyrus (Xing et al., 2022).

Overall, in studies reporting an association with WMH, no study reported global LFO changes, or changes in LFOs by tissue type and two studies reported no significant associations in LFOs between participants with WMHs and controls (Ding et al., 2015; Kim et al., 2020). Relative changes in LFOs in ROIs showed some consistency between studies, including increased LFOs in the deep grey structures and decreased LFOs in the parietal lobe, occipital lobe, and cerebellum (Table 1).

### Other manifestations of sporadic cSVD

LFOs were reported in 1 study in 66 patients with cerebral microbleeds versus 36 controls, with increased ALFF in the right insula, putamen, and left precuneus and decreased fALFF in the right precentral gyrus and postcentral gyrus (Feng et al., 2021). No global or tissue-specific values were noted. In 48 patients with lacunar stroke versus 28 controls, LFOs were increased in the temporal lobe and decreased in the precuneus, cuneus, and frontal lobe (Ni et al., 2016). No global or tissue-specific differences were reported. No papers were found that described LFOs and enlarged perivascular spaces or small subcortical infarcts and met the eligibility criteria.

### Multiple imaging markers of cSVD

Four papers included participants who had multiple imaging markers of cSVD. In 26 patients with either moderate–severe WMHs and/or multiple lacunar infarcts in the periventricular and deep white matter structures and 28 age-matched controls ALFF was increased in the right hippocampus, right thalamus, and the posterior part of the default mode network and decreased in the anterior part of the default mode network (Yi et al., 2012). They did not report any global or tissue-specific differences.

Li et al. analysed the ALFF of 30 patients with subcortical ischemic vascular disease (SIVD), encompassing lacunar infarcts and WMHs, and 35 controls (Li et al., 2014). No global or tissue-specific values were reported but the ALFF was increased in the bilateral anterior cingulate cortex, right putamen, and right supplementary motor area and decreased in the right precuneus and right angular gyrus. These differences were noted in the frequency band 0.01–0.073 Hz.

Only one study analysed LFOs in the visual cortex of 13 patients with imaging markers of cSVD by NIRS versus 14 healthy controls (Schroeter et al., 2005). Participants’ LFOs were impaired within the frequency range 0.07–0.12 Hz but unaltered within 0.01–0.05 Hz.

### CADASIL

One paper described the relationship between ALFF values in 22 patients with CADASIL and 44 matched controls (Su et al., 2019). No global or tissue-specific differences were reported. However, patients with CADASIL had higher ALFF in the bilateral superior frontal gyrus and left cerebellar anterior and posterior lobes and significantly lower values in the right precuneus and cuneus.

## Discussion

We identified 15 studies describing the relationship between LFOs and imaging measures of cSVD, of which eight were published in the last 3 years. No study reported global changes in LFOs, reflecting systemic influences on cerebral blood flow, or by specific tissue type, and no study aimed to differentiate LFOs due to vascular or neuronal function. However, despite marked variation in the direction of effect in anterior cortical regions in these predominantly small, low-quality studies, there were some consistent regional changes associated with cSVD. LFOs in the posterior cortex were reduced, whilst there were increased LFOs in the temporal lobes and deep grey nuclei in participants with cSVD.

LFOs have principally been investigated as a marker of neuronal activity in Parkinson’s disease (Yue et al., 2020), mild cognitive impairment (Xi et al., 2012; Pan et al., 2017), and dementia (Yang et al., 2018), and it has been proposed that altered amplitude of LFOs could mediate effects on cognition by tests such as the MoCA or trail-making test (Su et al., 2019; Xing et al., 2022). Although, LFOs have been found to have a major vascular contribution in healthy participants (Tong et al., 2019), with physiological time delays in the signal moving across the brain (Tong et al., 2017), consistent with the signal in the carotid arteries (Schreiber et al., 2002), and the peripheries (Li et al., 2018), we found no studies specifically assessing change in LFOs of vascular origin in participants with cSVD. Furthermore, as most studies in this review normalised LFOs by the global ALFF and did not report global or tissue-specific LFOs, they will have attenuated the effect of systemic vascular dysfunction in patients with cSVD, evident in the LFO signal, whether due to widespread endothelial dysfunction or altered transmission of systemic blood pressure fluctuations to the cerebral circulation. Indeed, some authors noted that they deliberately chose the frequency in which they measured LFOs to exclude potential physiological contributions to the LFO signal (Li et al., 2014; Feng et al., 2021).

Local differences in LFOs in patients with cSVD may reflect focal neuronal dysfunction but are also likely to provide a measure of vascular function. Differences in LFOs were seen in the deep grey matter and archicortex which may reflect systemic vascular function in these regions more exposed to systemic vascular haemodynamics compared to reduced function in the cortex, which could reflect either neuronal dysfunction or local endothelial dysfunction. However, in the absence of consistent results in other cortical regions further larger and more rigorous studies will be required to determine the vascular contribution to LFOs by tissue type.

LFOs present up to 0.1 Hz have been demonstrated in blood flow-dependent signals throughout the body, including the brain (Tong and Frederick, 2014). These are strongly dependent upon sympathetic autonomic function in studies of heart rate variability, baroreceptor sensitivity, and in measures of cerebral autoregulation using transcranial ultrasound of large intracranial vessels (deBoer et al., 1987; Cooley et al., 1998; Julien, 2006). This autonomic control of regional blood flow is likely mediated by endothelial control of the underlying smooth muscle, and therefore alterations in LFOs may reflect both autonomic control and endothelial dysfunction. Similarly, cSVD is strongly associated with systemic endothelial dysfunction including hypertension, diabetes, and increased arterial stiffness (Webb et al., 2012), and intracerebrally with endothelial dysfunction of cerebral small vessels. This may be either due to long-standing hypertension or a primary endotheliopathy that causes impaired regulation of cerebral blood flow and results in both SVD and systemic hypertension (Wardlaw et al., 2013a). Endothelial dysfunction in SVD is manifest as impaired reactivity of cerebral small vessels to external stimuli, such as carbon dioxide (Stevenson et al., 2010), breakdown of the blood–brain barrier (Wardlaw et al., 2016), and impaired autoregulation of cerebral blood flow (Markus, 2008). Whether endothelial dysfunction and loss of local blood flow regulation reflects systemic haemodynamic factors or is driven by a primary endotheliopathy, it is a core index of early cSVD pathology and is likely central to the underlying pathophysiological process. If LFOs are associated with endothelial dysfunction then they may provide a practical method to assess endothelial function and autonomic-dependent control of cerebral blood flow in patients (Ma et al., 2020).

One source of heterogeneity in the direction of association between LFOs and cSVD may reflect differences in acquisition and analysis methods. Although these were relatively consistent for some parameters (e.g., use of ALFF for the analysis), these were optimised for neuronal contributions without clear justification for the choices made. Normalisation of the ALFF by global values, performed in most studies, limits the ability to detect overall change in ALFF amplitude by tissue-type, due to the influence of systemic oscillations, resulting in studies that can only measure relative cortical differences. Furthermore, sampling must be performed at a rate of at least twice the highest frequency that is to be detected (Shannon, 1949) and therefore longer scan acquisition repetition times (TR) may result in aliasing of the cardiac and respiratory signal (Huotari et al., 2019). Other pre-processing techniques such as spatial smoothing or principal component analysis, which is often intended to remove physiological signal, may lead to loss of signal that contributes to LFOs (Tong et al., 2013; Caparelli et al., 2019). Finally, the majority of studies focused on LFOs within 0.01–0.08 Hz which may not include important changes around the 0.1 Hz frequency peak. One study found that spontaneous LFOs in the 0.07–0.12 Hz were specifically impaired in cSVD, in contrast to spontaneous very-low frequency oscillations in the band 0.01–0.05 Hz (Schroeter et al., 2005).

There are limitations in this review. Although all studies were systematically assessed for quality, this metric showed that the studies were highly variable, with the majority of studies being cross-sectional in design, with small numbers of participants, and of weak-to-moderate quality on the NIH Quality Assessment Tool. A significant proportion of reported studies were only available as abstracts or in languages not available for translation. The majority of studies did not report global or tissue-specific measures of LFOs, preventing a more objective analysis of the potential vascular contribution to LFOs. Even for regional changes in LFOs, there was limited and heterogeneous data, with marked variation in patient selection, image acquisition, and processing, such that a meta-analysis was not possible. Finally, there were too few studies assessing LFOs in markers of cSVD other than WMHs to determine any consistency of effects.

**Figure 4:**
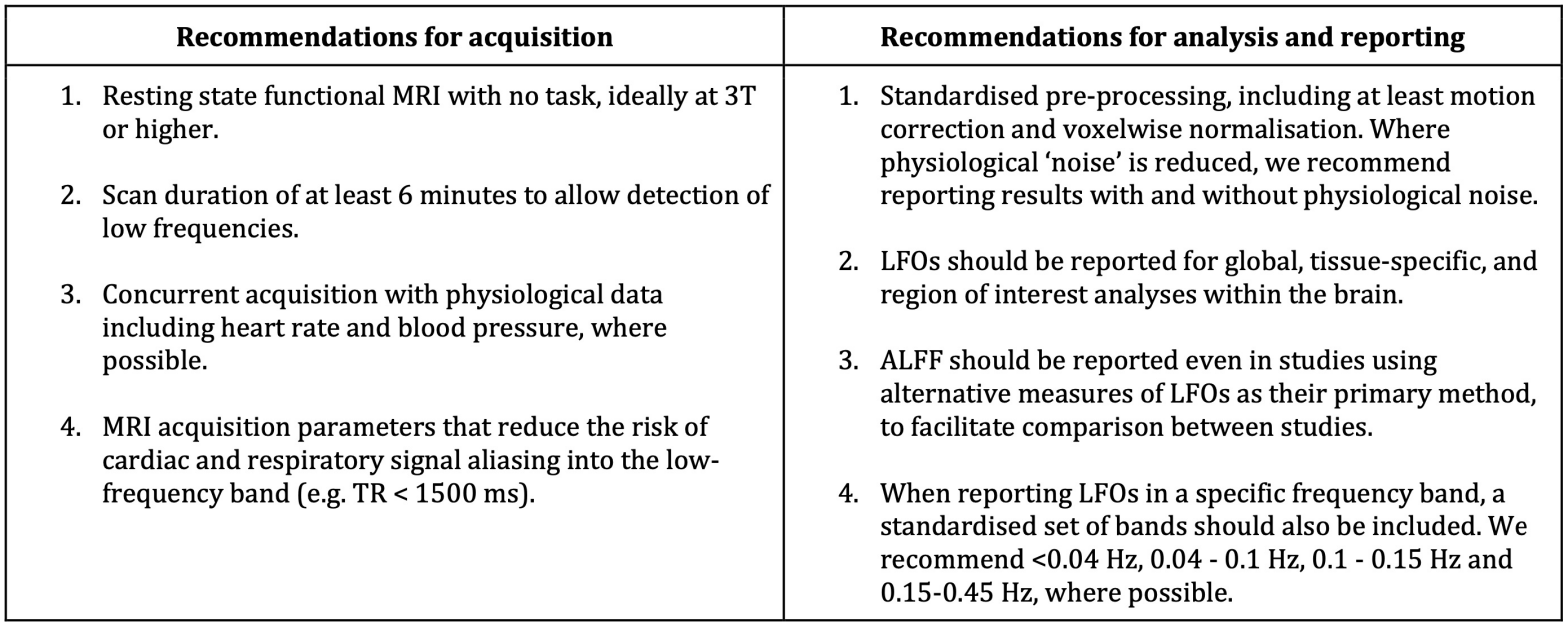
Proposed standards for studies measuring LFOs in study participants.

Since no studies in this systematic review noted global or tissue-specific differences in LFOs, future studies may benefit from reporting these values to help disentangle the neuronal from vascular low-frequency signals, as well as utilisation of more advanced methods with the capacity to discriminate vascular-from neuronal-dependent signals (e.g., Rapitide software; Frederick et al., 2012). As neuronal activity is not dominant in the white matter, measuring and reporting LFOs in this tissue will likely better reflect the vascular component of LFOs. Consequently, comparing any differences between LFOs in the grey and white matter may help researchers better understand neuronal versus haemodynamic contributions to the LFO signal. In addition, studies with follow-up scans would be useful to evaluate if LFOs change over time in patients with cSVD. Studies with larger numbers of participants would better ensure that findings are statistically significant and more representative of the population, for example by taking advantage of open access data sets (UK Biobank, Human Connectome Project). However, critically, a consensus is required for the acquisition, measurement and analysis of LFOs, both as a marker of neuronal function and as a measure of vascular function, as well as reporting standards for future publications in this field. We have proposed eight standards for future studies as a result of this review (Figure 4). Acquisition should ideally be conducted at resting state with a 3 T or higher strength MRI scanner due to its improved signal-to-noise ratio (SNR), spatial resolution, and increased sensitivity to the BOLD signal (Raimondo et al., 2021). A suggested minimum of 6 min acquisition time will increase the amount of data collected and enhance the SNR (Van Dijk et al., 2010). A TR < 1,500 ms will reduce the potential for cardiac and respiratory signal aliasing into the low-frequency band (Huotari et al., 2019). Pre-processing that uses principal component analysis or spectral filters may inadvertently also remove non-nuisance signal that contributes to LFOs (Tong et al., 2013). Consequently, limiting pre-processing to minimal and standardised pipelines, which may include motion correction and voxel-wise normalisation, will help to reduce the risk of losing signal of interest. Concurrent acquisition of heart rate and blood pressure will help ensure vascular signal that may contribute to LFOs is not lost before analysis (Chang et al., 2013; Whittaker et al., 2019). Finally, reporting of LFOs, including ALFF, in both global and tissue-specific regions for a standardised set of frequency bands, will allow for comparison across studies and detection of variations in LFOs among different tissues and frequencies.

## Conclusion

This systematic review has shown that there is recent and increased interest in the use of LFOs as an index for cSVD. However, there is little consensus on the parameters for acquisition and measurement. The majority of studies are small and cross-sectional in design and of varying quality. Furthermore, most studies view LFOs as a surrogate for neuronal activity, focusing exclusively on grey matter, despite evidence for a significant haemodynamic component of LFOs in other studies in the wider literature. As such, future research should assess both the neuronal and vascular contribution to LFOs throughout the brain, with the potential for an easily measurable and reliable marker of underlying vascular dysfunction in cSVD.

## Data availability statement

The original contributions presented in the study are included in the article/Supplementary material, further inquiries can be directed to the corresponding author.

## Author contributions

JT: Writing – original draft, writing – review and editing. PJ: Writing – original draft, writing – review and editing. AW: Writing – original draft, writing – review and editing.

## Funding

This work was supported by the Wellcome Trust Grant 206589/Z/17/Z.

## Conflict of interest

The authors declare that the research was conducted in the absence of any commercial or financial relationships that could be construed as a potential conflict of interest.

## Publisher’s note

All claims expressed in this article are solely those of the authors and do not necessarily represent those of their affiliated organizations, or those of the publisher, the editors and the reviewers. Any product that may be evaluated in this article, or claim that may be made by its manufacturer, is not guaranteed or endorsed by the publisher.
