# Supplementary figures and images for: Low-frequency oscillations in the brain show differential regional associations with severity of cerebral small vessel disease: a systematic review
Source: Front Neurosci. 2023 Aug 31;17:1254209. doi: 10.3389/fnins.2023.1254209 (PMC10501452; doi:10.3389/fnins.2023.1254209)

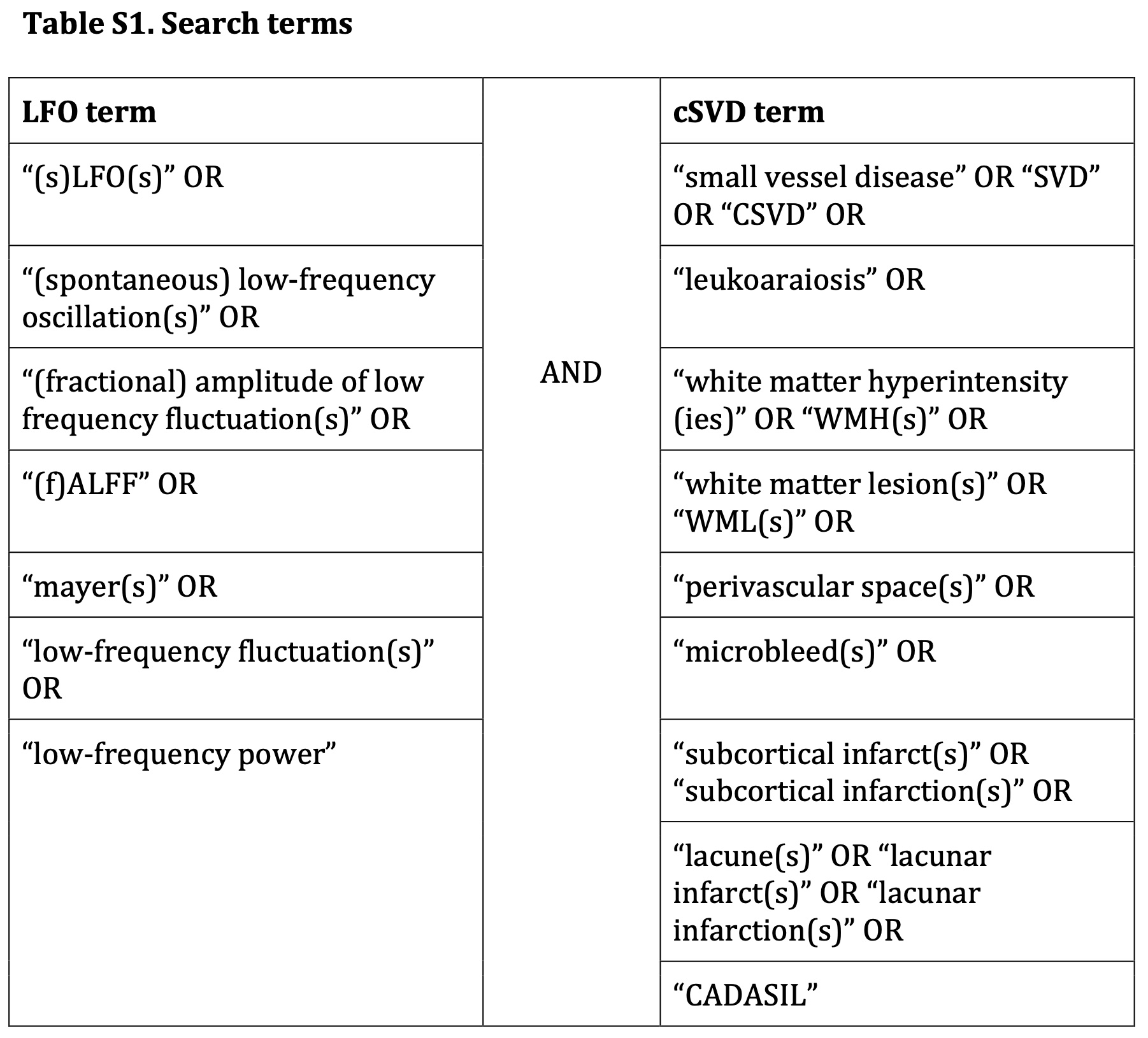

Supplement: Supplementary file 1 [file Image_1.JPEG]

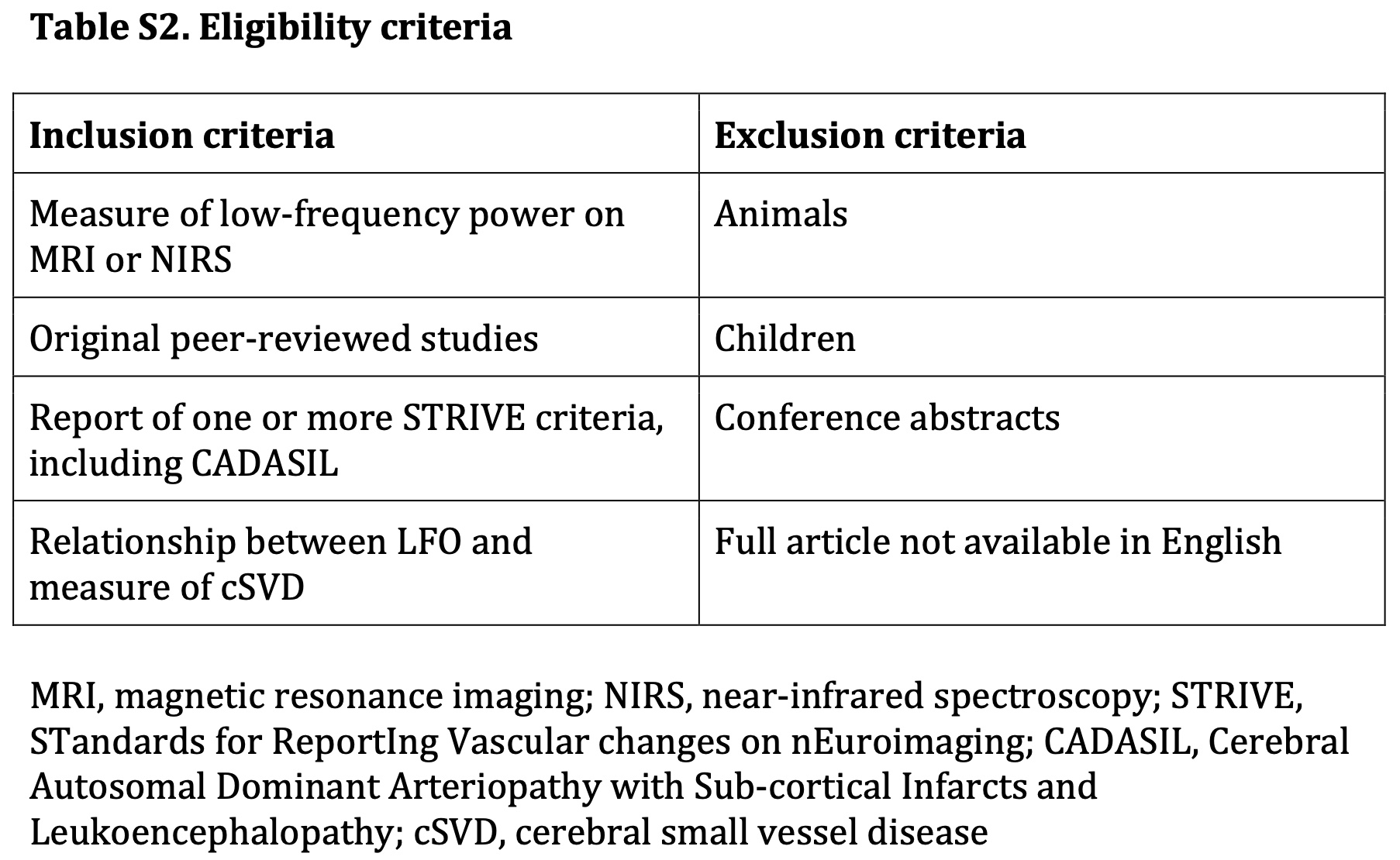

Supplement: Supplementary file 2 [file Image_2.JPEG]

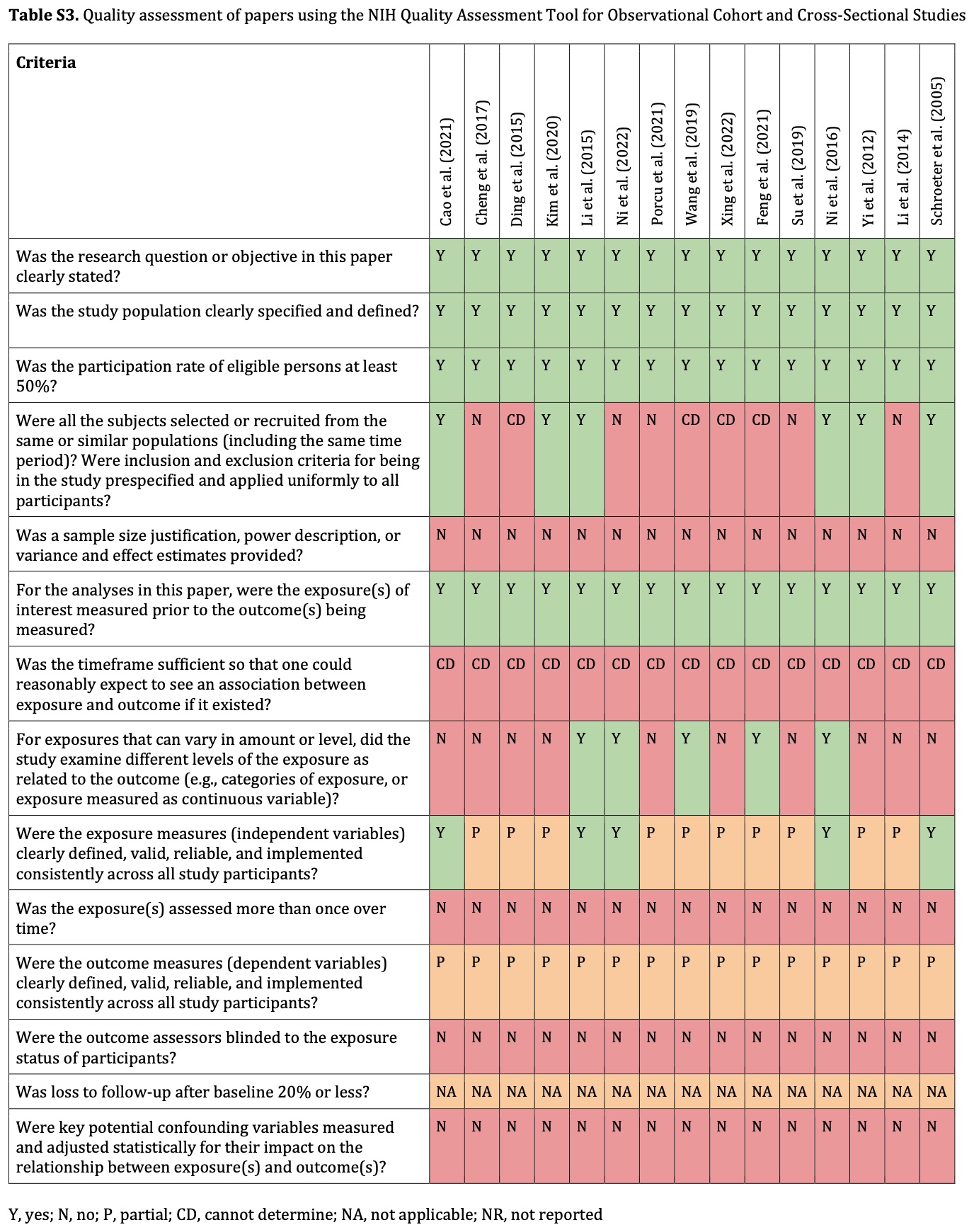

Supplement: Supplementary file 3 [file Image_3.jpg]
